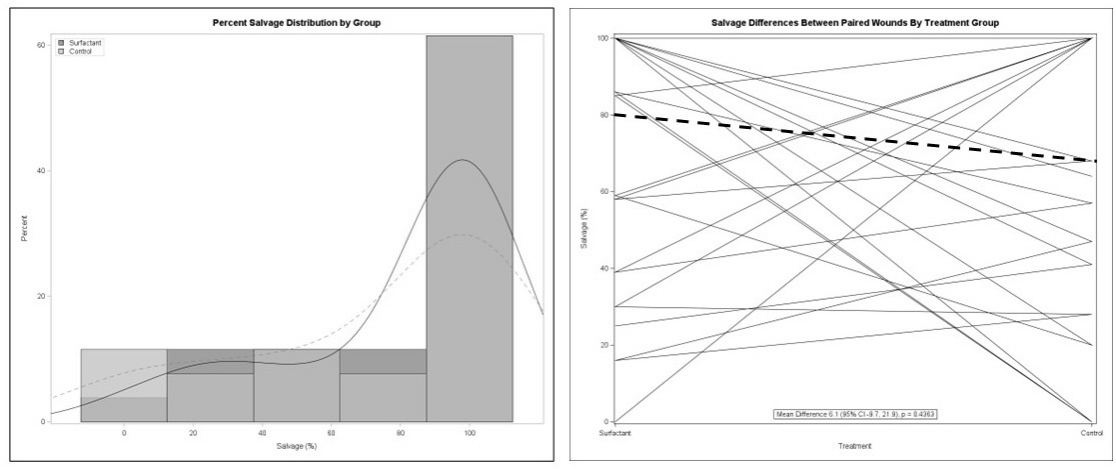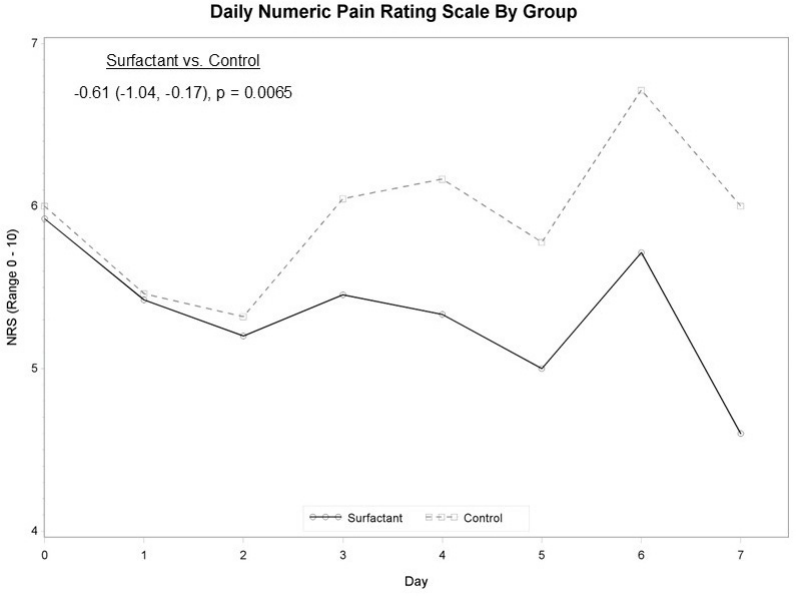# 535 Double-blind, Randomized, Controlled Trial Evaluating Early Application of a Surfactant-based Dressing for Partial-thickness Burns (EARLY)

**DOI:** 10.1093/jbcr/iraf019.164

**Published:** 2025-04-01

**Authors:** David Hill

**Affiliations:** Regional One Health

## Abstract

**Introduction:**

Surfactant-based wound dressings (WSD) have been utilized in chronic, non-healing wounds and small burn wounds to soften and aid removal of wound debris. We conducted a double-blind, randomized controlled study evaluating paired, non-contiguous partial thickness burn wounds comparing WSD versus bacitracin. We hypothesized early application of WSD would lead to less wound conversion and less painful wound care.

**Methods:**

Sample size was determined based on a projected 25% improvement in salvaged tissue (i.e., excised tissue) with bacitracin as the comparator. Wounds were paired; two non-contiguous partial thickness areas less than 10% TBSA each. Wound care was performed daily, per protocol. Additional outcomes included grafted area, infection, and procedure-associated pain. Patients and assessing surgeons were blinded to treatment assignment. Paired analysis performed for primary outcome and mixed modeling for change in Numeric Pain Rating Scale. SAS 9.4 was used for analysis.

**Results:**

Patients were consented and treated within 24 hours of injury. All cases were deemed initially necessary for admission. Patients were followed until 95% re-epithelialization occurred or were discharged. Vancouver scar scale was utilized post discharge. Twenty-seven patients were consented. The average time from injury to consent was 14.6 ± 5.6 hours. The average age was 40.9 ± 16.5 years with 65% being male, 50% Black, and 50% Caucasian. Flame injury was most common. The average total body surface area burned was 8.8 ± 3.6%. There was no difference in percent of salvaged tissue, calculated as the amount excised (cm2) relative to the original size of injury [6.1% (95% CI -9.7, 21.9), p=0.4363); 26.9% versus 38.5% of patients had their wounds convert to a deeper injury. However, there was a significant difference [-0.61 (95% CI -1.04, -0.17), p=0.0065) in patient reported pain between treatment assignment (measured at baseline and after each wound care session). There was one wound infection in each group.

**Conclusions:**

WSD facilitates easier debridement, is resistant to infection without need of antimicrobial exposure, and shows improved patient tolerability.

**Applicability of Research to Practice:**

WSD is a useful dressing that can be easily applied early after injury and can improve patient’s wound care experience.

**Funding for the Study:**

Medline funded this study through an investigator-initiated grant, but has not reviewed the results.